# Predicting autism diagnosis through intellectual ability. A machine-learning model

**DOI:** 10.1192/j.eurpsy.2026.10199

**Published:** 2026-07-31

**Authors:** K. Lucci, D. Kandaleft

**Affiliations:** 1 Department of Biological Psychology, SWPS University; 2 CDT Spektrum, Warsaw, Poland; 3 Dr Dona Psychology Clinic, Dubai, United Arab Emirates

## Abstract

**Introduction:**

Autism spectrum disorder (ASD) is a pervasive neurodevelopmental condition characterized by social, communication, and sensorimotor difficulties. Traditional diagnostic procedures such as ADOS-2 are effective but time-consuming, costly, and reliant on highly trained professionals. Machine learning (ML) offers opportunities for more efficient and objective approaches. Intellectual functioning has historically been linked to ASD, yet its diagnostic role remains unclear.

**Objectives:**

This study aimed to evaluate whether cognitive profiles measured with the Stanford-Binet Intelligence Scales, Fifth Edition (SB-5), can predict ICD-10 ASD subtypes using ML models.

**Methods:**

A total of 68 children with ASD (51 males, 17 females; age range 2–17 years, M = 8.9, SD = 3.8) completed the SB-5. ICD-10 classifications included F84.0 (n = 11), F84.1 (n = 21), and F84.5 (n = 36). A control group of non-ASD children referred for IQ testing was used for comparison. Eight ML algorithms were tested: k-nearest neighbors, support vector machines (linear and RBF), decision tree, random forest, logistic regression, neural network, and a majority voting ensemble. Hyperparameters were tuned via grid search with nested 5-fold cross-validation, and models were evaluated using 10-fold cross-validation. Accuracy, precision, recall, and F1 score were computed. Analyses were repeated controlling for age.

**Results:**

ASD subgroups differed significantly in intellectual functioning. Children with F84.5 had higher mean IQ scores (M = 102.1, SD = 12.7) compared with F84.0 (M = 78.1, SD = 14.5) and F84.1 (M = 71.2, SD = 18.9; p < .001). Among ML models, logistic regression achieved the best performance (75% accuracy), followed closely by random forest and majority voting (74%). Controlling for age increased accuracy up to 79%. Predictions for F84.1 vs. F84.5 reached 88% accuracy, whereas F84.0 proved more difficult to classify, likely due to small sample size and heterogeneity.

**Conclusions:**

Machine learning applied to standard cognitive test data can distinguish ICD-10 ASD subtypes with promising accuracy. Logistic regression and ensemble models were most effective, suggesting interpretable approaches may have clinical utility. The predictive role of intellectual ability supports its relevance in diagnostic frameworks, though further validation in larger, diverse samples is required. Integrating ML with cognitive assessments could contribute to faster, more cost-effective, and objective ASD diagnostics.

**Disclosure of Interest:**

None Declared

